# Reduction of T Lymphoma Cells and Immunological Invigoration in a Patient Concurrently Affected by Melanoma and Sezary Syndrome Treated With Nivolumab

**DOI:** 10.3389/fimmu.2020.579894

**Published:** 2020-09-25

**Authors:** Maria Grazia Narducci, Anna Tosi, Alessandra Frezzolini, Enrico Scala, Francesca Passarelli, Laura Bonmassar, Alessandro Monopoli, Maria Pina Accetturi, Maria Cantonetti, Gian Carlo Antonini Cappellini, Federica De Galitiis, Antonio Rosato, Mario Picozza, Giandomenico Russo, Stefania D’Atri

**Affiliations:** ^1^Istituto Dermopatico dell’Immacolata, IDI-IRCCS, Rome, Italy; ^2^Department of Surgery, Oncology and Gastroenterology, Oncology and Immunology Section, University of Padua, Padua, Italy; ^3^Department of Hematology, University of Rome Tor Vergata, Rome, Italy; ^4^Veneto Institute of Oncology IOV-IRCCS, Padua, Italy; ^5^Laboratory of Neuroimmunology, IRCCS Fondazione Santa Lucia, Rome, Italy

**Keywords:** cutaneous T-cell lymphoma, PD-1 blockade therapy, immune sub-populations, Ki67 proliferation index, granzyme B

## Abstract

Despite the recent availability of several new drugs in hemato-oncology, T-cell lymphomas are still incurable and PD-1 blockade could represent a therapeutic chance for selected patients affected by these malignancies, although further studies are required to understand the biological effects of anti-PD-1 mAbs on neoplastic T-cells and to identify biomarkers for predicting and/or monitoring patients’ response to therapy. Sezary Syndrome (SS) represents a rare and aggressive variant of cutaneous T cell lymphoma (CTCL) with a life expectancy of less than 5 years, characterized by the co-presence of neoplastic lymphocytes mainly in the blood, lymph nodes and skin. In this study we analyzed longitudinal blood samples and lesional skin biopsies of a patient concurrently affected by SS and melanoma who underwent 22 nivolumab administrations. In blood, we observed a progressive reduction of SS cell number and a raise in the percentage of normal CD4+ and CD8+ T cells and NK cells over total leukocytes. Eight weeks from the start of nivolumab, these immune cell subsets showed an increase of Ki67 proliferation index that positively correlated with their PD-1 expression. Conversely, SS cells displayed a strong reduction of Ki67 positivity despite their high PD-1 expression. On skin biopsies we observed a marked reduction of SS cells which were no more detectable at the end of therapy. We also found an increase in the percentage of normal CD4+ T cells with a concomitant decrease of that of CD8+ and CD4+ CD8+ T cells, two cell subsets that, however, acquired a cytotoxic phenotype. In summary, our study demonstrated that nivolumab marked reduced SS tumor burden and invigorated immune responses in our patient. Our data also suggest, for the first time, that Ki67 expression in circulating neoplastic and immune cell subsets, as well as an enrichment in T cells with a cytotoxic phenotype in lesional skin could be valuable markers to assess early on treatment SS patients’ response to PD-1 blockade, a therapeutic strategy under clinical investigation in CTCL (ClinicalTrials.gov NCT03385226, NCT04118868).

## Introduction

Immune checkpoint (IC) inhibition with anti-PD-1 monoclonal antibodies (mAbs) represents a first-line standard treatment for metastatic melanoma, producing objective response rates of 30–40% and significantly increasing progression free survival and overall survival (1). Anti-PD1 mAbs have also been approved for the treatment of other solid tumors (2), classical Hodgkin lymphoma, and have shown some efficacy in a number of other B-cell malignancies (3). Instead, the therapeutic potential of PD-1 blockade in T-cell malignancies is still uncertain, even if clinical benefits have been reported by a number of studies (4–7). The blockade of PD-1/PD-L1 axis in T-cell disorders poses an unique challenge, since PD-1 represents a tumor suppressor in T cells and its inhibition can potentially promote lymphomagenesis (8). Notably, development of a secondary T-cell neoplasia in patients subjected to anti-PD-1 therapy for their primary tumor has been described (9–11). Moreover, the development of a T-cell lymphoma was found to be an adverse effect of IC inhibitors with an incidence of 0.02% (11). These findings highlight the need to gain a better understanding of the biological effects of anti-PD-1 mAbs in T-cell malignancies.

With this aim, we describe here the case of a female patient with synchronous metastatic melanoma and Sezary syndrome (SS), an aggressive leukemic variant of cutaneous T-cell lymphoma (CTCL), (12, 13) who was treated with nivolumab for her melanoma and showed a remarkable clinical and biological response of SS. We report the changes occurring in phenotype and/or frequency of circulating and skin-resident SS cells and immune sub-populations during therapy, which suggest that their monitoring could help to determine SS patient’s clinical response early on treatment.

## Case Report

A 72-year-old Caucasian female come to our observation in January 2017 due to the appearance of erythrodermia and severe itching. Physical examination evidenced erythematous lesions of bright red color involving about 70% of the skin. During the same visit, a pigmented lesion of about 1.5 cm in diameter, highly suspected of being a melanoma, was observed on the scalp vertex. This lesion and a portion of erythrodermic cutis on the left arm were excised for histological analyses. The lesion of the scalp was diagnosed as an ulcerated nodular melanoma (17 mm Breslow, mitotic rate >1 mitosis/mmq, pT4b, UICC 2009). BRAF mutational analysis by Cobas^®^ 4800 and subsequent Sanger identified the V600E mutation. Histological examinations of the erythrodermic cutis specimen and the assessment of TCR clonality by PCR-BIOMED2 (14) diagnosed an epidermotropic T-cell lymphoma. Immunophenotyping of peripheral blood mononuclear cells (PBMCs) by flow cytometry identified lymphocytes with the CD3+/CD4+/CD7−/CD26− phenotype and a CD4+/CD8+ ratio of 16. Clonality analysis showed an expansion of 95% of CD4+ lymphocytes with rearrangement of TCR-Vβ 5.1, calculated on all CD4+ lymphocytes. Subsequent total body computed tomography (TC) and positron emission tomography (PET)/TC evidenced bilateral cervical, axillary and inguinal lymphadenopathy with pathological hyperfixation. The patient underwent enlargement of the previous exeresis in the vertex region, and right laterocervical lymphadenectomy which identified one lymph node positive for melanoma. Patient was diagnosed as having non-operable stage IIIc melanoma and stage IVAi SS (15). The patient had no family history of melanoma or other neoplasia.

On July 2017, the patient started a first-line therapy with nivolumab (3 mg/kg, 1-h infusion, every 2 weeks), which was carried out for 22 administrations, i.e., until therapy switching to dabrafenib (150 mg BID) plus trametinib (2 mg/die) for brain metastases not responding to stereotactic radiotherapy. Targeted therapy continued for 6 months, until patient death for melanoma. Melanoma best response to nivolumab and targeted therapy was partial response, according to irRECIST (16) and RECIST 1.1 (17) criteria, respectively. Patients also achieved partial response of SS, according to consensus global response criteria (18). In particular, physical examination after 6 nivolumab administrations evidenced a substantial reduction of erythroderma and itching which persisted up to patient death for melanoma.

Immune-related adverse effects (AEs) were represented by a grade-1 thyroiditis, and vitiligo-like lesions on face, body, upper and lower limbs, which became evident after 5 nivolumab administrations and further increased during therapy.

## Materials and Methods

### Blood Sampling

Peripheral blood (PB) was collected before the 1st nivolumab administration and 15 days after selected administrations, namely the 1st, 2nd, 4nd, 6th, 9th, 12th, 17th, and 21st, which corresponded to baseline (T0) and to 2, 4, 8, 12, 18, 24, 34, and 42 weeks from the start of nivolumab, respectively (hereafter referred to as T0, T2, T4, T8, T12, T18, T24, T34, and T42). An additional blood sample was collected after the 22nd nivolumab administration (T44) and 16 weeks of targeted therapy (T60).

Experimental procedures conducted for flow cytometry, immunohistochemistry (IHC) and multiplex fluorescence IHC (mIHC) analyses are described in Supplementary Material.

## Results

### PB Changes in Immune Cell Subsets and Tumor Burden During Nivolumab Therapy

**FIGURE 1:**
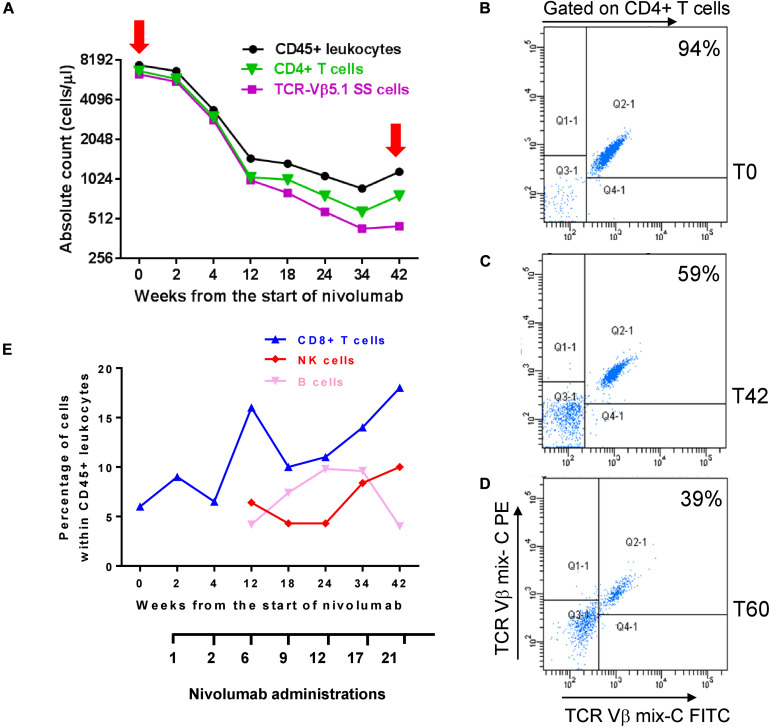
Changes of circulating SS cells and immune cell subsets during nivolumab treatment. **(A)** Absolute counts of total CD45+ leukocytes, CD4+ T cells and SS cells were determined at T0 and the indicated weeks from the start of nivolumab, as described under section “Materials and Methods.” **(B–D)** PBMC were co-stained with anti-TCR-Vβ 5.1 (mix **C**) and anti-CD4 mAbs at T0, T42 [red arrows in graph **(A)**] and T60. Percentage of SS cells was evaluated in pre-gated CD4+ T cells and is showed into the plots. **(E)** Percentages of CD8+ T cells, NK and B cells were calculated within total CD45+ leukocytes.

To investigate how nivolumab influenced PB lymphocyte sub-populations and tumor burden, we determined the counts/ml of CD45+ leukocytes, CD4+ SS cells, and total CD4+ and CD8+ T cells at T0 and after selected nivolumab administrations up to 42 weeks (T42) from the start of therapy. Moreover, we monitored the counts/ml of CD16+ CD56+ NK cells and CD19+ B cells at T0 and from T12 to T42. The counts/ml of SS cells was calculated from the percentage of TCR-Vβ 5.1+ SS cells detected within CD4+ lymphocytes (Figures 1A,B).

As shown in Figures 1A,B, we measured an absolute count of 6.4 × 10^3^/ml neoplastic cells at T0, representing 94% of CD4+ T cells (6.8 × 10^3^/ml) and 85% of CD45+ leukocytes (7.5 × 10^3^/ml). All these three populations decreased maintaining a similar ratio until T4 (Figure 1A). After this point, we observed a more consistent reduction of neoplastic cells that at T42 were 0.45 × 10^3^/ml, representing 59% of CD4+ T cells (0.77 × 10^3^/ml) and 38% of CD45+ leukocytes (1.1 × 10^3^/ml) (Figures 1A,C).

Notably, evaluation of neoplastic cells at T60 revealed a still low tumor burden with 0.7 × 10^3^/ml SS cells representing 39% of CD4+ T cells (2 × 10^3^/ml) (Figure 1D) and 16% of CD45+ leukocytes (4.4 × 10^3^, data not shown).

Nivolumab also induced changes in the percentage of CD8+ T cells, NK cells and B cells within CD45+ leukocytes (Figure 1E). CD8+ T cells increased from 6% detected at T0 to 18% detected at T42, with a spike of 16% observed at T12. This trend was also supported by the decreasing ratio of CD4+/CD8+ T cells measured from T0 to T42 (Supplementary Table 1).

A raise of NK cells from 1.77% at T0 (not shown in figure) to 6.4% at T12 and 10% at T42 was also observed, as well as a variation in the percentage of CD19+ B cells which displayed a bell-shaped curve, starting from 0.58% at T0 (not shown in the figure), progressively increasing up to 9.6% from T12 to T24, and returning to 4% at T42 (Figure 1E).

### Expression of PD-1 in SS Cells and Normal Immune Cell Sub-Populations and Their Invigoration Response to Nivolumab Therapy

**FIGURE 2:**
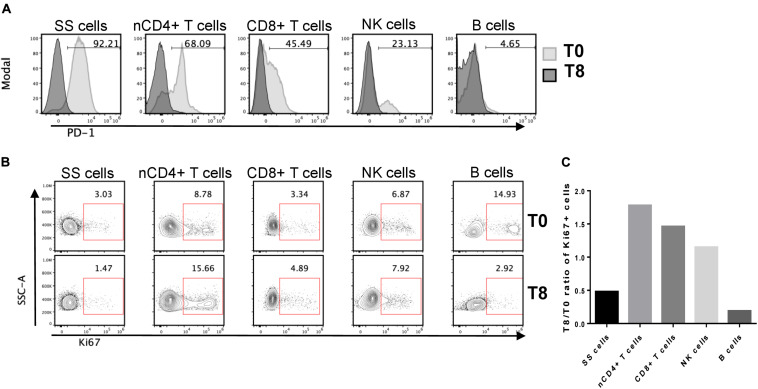
PD-1 expression in SS cells and normal immune cell subsets and relative patterns of Ki67+ cell frequencies. **(A)** Frozen PBMCs from SS patient collected at T0 and T8 were thawed and stained for flow cytometry. Pre-gated live single SS cells, normal (n) CD4+ and CD8+ T cells, CD16+ NK cells and CD19+ B cells (see Supplementary Figure 1 for the gating strategy) were inspected for PD-1 expression by overlaying T0 vs. T8 histograms. Numbers inside plots indicate the percentage of PD-1+ cells at T0. **(B)** Ki67 expression patterns in the same cell subsets defined in **(A)**. The percentage of Ki67+ cells is indicated by the numbers inside the plots. **(C)** The bar chart shows the T8/T0 ratios of Ki67+ cell frequencies for the indicated sub-populations.

To better understand the therapeutic effect of nivolumab on SS, we evaluated PD-1 expression in neoplastic cells and immune sub-populations by flow cytometry using PBMCs collected at T0 and T8. Using the gating strategy shown in Supplementary Figure 1, we found that PD-1 was expressed by 92% of SS cells, 68% of normal CD4+ T cells, 45% of CD8+ T cells, 23% of NK cells and only 4.6% of B cells (Figure 2A). None of these cell subsets showed PD-1 expression at T8, accordingly with PD-1 receptor occupancy by nivolumab which prevents the binding of the anti-PD-1 mAb used for staining (19) (Figure 2A).

These results pointed out that both SS cells and healthy immune sub-populations are targets of nivolumab and can be potentially unlocked by this therapy in terms of proliferation and/or activity. We thus measured the amount of proliferation of each cell subset after nivolumab treatment. PBMCs collected at T0 and T8 were stained with mAbs against lymphoid lineage markers and Ki67, a nuclear proliferation marker (20).

As showed in Figures 2B,C normal CD4+ T cells were the most responsive to nivolumab, showing an increase of 1.8-fold in the percentage of Ki67+ cells at T8 with respect to T0. CD8+ T cells and NK cells displayed an increase of proliferation of 1.5-fold and 1.2-fold, respectively. Conversely, SS cells and B cells displayed a marked reduction of proliferation, showing a T8/T0 ratio of Ki67+ cell frequencies of 0.48 and 0.19, respectively.

PD-1 expression follows a tri-modal pattern in T cells that can be classified as PD-1 negative, PD-1^*low*^ and PD-1^*high*^ expressing sub-populations (21).

A closer look at these PD-1 expression-related subsets at T0 within normal CD4+ and CD8+ T cells demonstrated that PD-1^*high*^ cells displayed the highest expression of the activation/proliferation markers Ki67, CD71, and HLA-DR supporting an ongoing immune response (22) (Supplementary Figure 2).

### Evaluation of Skin-Resident SS Cells and Tumor Infiltrating Lymphocytes (TILs) During Nivolumab Therapy

**FIGURE 3:**
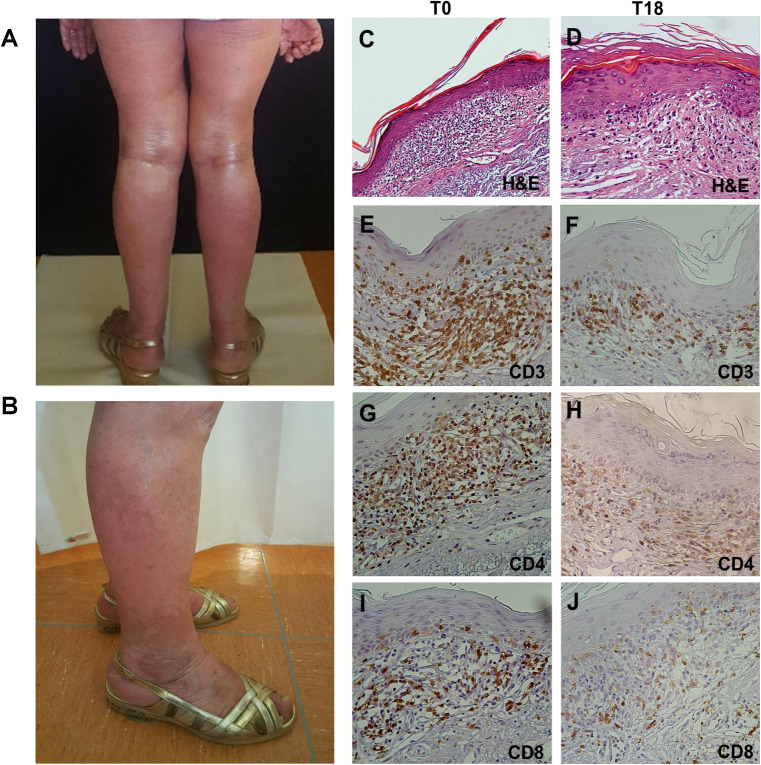
Clinical presentation and histopathological features of SS. **(A)** Diffuse erythroderma involving 70% of total body at T0. **(B)** Reduced erythroderma and presence of vitiligo-like lesion at T8. **(C–J)** Hematoxylin-eosin (H&E) staining and IHC on lesional skin biopsies. **(C)** H&E staining of T0 biopsy revealed a dense band of atypical T lymphocytes infiltrating papillary dermis (magnification x10/0.30NA).**(D)** H&E staining of T18 biopsy revealed a reduced neoplastic infiltrate with a lichenoid aspect (magnification x20/0.40NA). **(E–J)** IHC analysis for CD3+, CD4+, and CD8+ cells showed a reduction of their density from T0 to T18 (magnification x20/0.40NA).

Histopathological analysis performed on lesional skin biopsies revealed a dense band of atypical T lymphocytes infiltrating papillary dermis at T0, that appeared reduced and lichenoid at T18. Immunohistochemistry (IHC) detection of CD3+, CD4+, and CD8+ T cells evidenced a marked reduction of their density from T0 to T18 (Figure 3).

**FIGURE 4:**
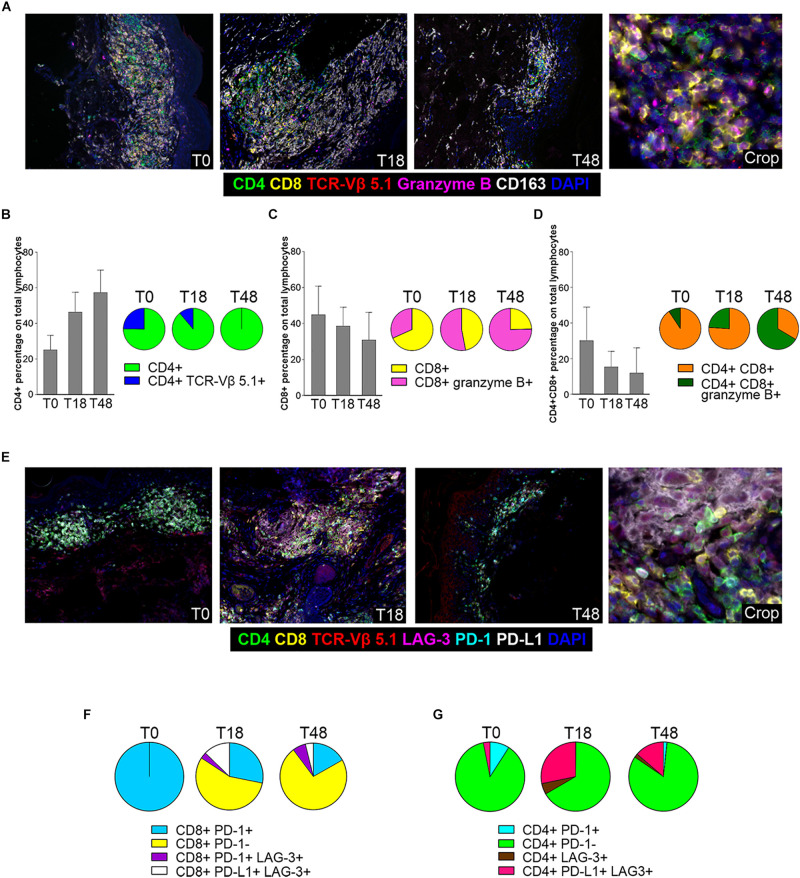
mIHC analysis of skin infiltrating SS cells and TILs. **(A)** Representative 7-color multispectral images of SS cells and TILs in lesional biopsies collected at T0, T18 and T48. Immune markers and color code are shown in the underlying legend. Original magnification X20. **(B–D)** Left: mIHC cell percentage of CD4+ **(B)**, CD8+ **(C)**, CD4+ CD8+ **(D)** cells calculated among total lymphocytes in biopsies collected at T0, T18 and T48. Data reported for each cell subset are the mean values and standard deviation (SD) of about 20 fields from the same sections. Right: pie charts of mIHC data from biopsies collected at T0, T18, and T48. Data reported for each cell subset are the mean values derived from the analysis of the same fields considered in the flanking histograms. **(E)** Representative 7-color multispectral images of SS cells and TILs in biopsies collected at T0, T18, and T48. Immune markers and color code are indicated in the underlying legend. Original magnification X20. **(F,G)** Pie charts of checkpoint molecule expression on CD8+ and normal CD4+ lymphocytes calculated in biopsies collected at T0, T18, and T48. Data reported for each cell subset are the mean values derived from the analysis of about 20 fields from the same sections.

Next, to better evaluate therapy-induced modulation of skin-resident SS cells and TILs, we used multiplex fluorescence IHC (mIHC) on T0, T18 and T48 skin biopsies (Figure 4). In accordance with IHC findings, a decreased of total lymphocyte density was observed from T0 to T18. At T48 (i.e., 4 weeks after therapy switching from nivolumab to dabrafenib + trametinib) a partial recovery of lymphocyte density was evidenced (Figure 4A and Supplementary Figure 3).

Assessment of CD4+ subsets revealed that percentage of normal CD4+ T cells (over total CD4+ and CD8+ T cells) gradually increased from 25 to 60%, from T0 to T48 (Figure 4B). Conversely, the percentage of CD4+ TCR-Vβ 5.1+ SS cells calculated within CD4+ lymphocytes, decreased from 25% at T0 to 11% at T18, to completely disappear at T48 (Figure 4B left). Moreover, at T18 we detected a small proportion of CD4+ lymphocytes endowed with a cytotoxic phenotype (CD4+ granzyme B+) that was no longer present at T48 (data not shown).

Infiltrating CD8+ lymphocytes showed an opposite trend of CD4+ lymphocytes. At T0, they represented 45% of total CD4+ and CD8+ lymphocytes and this percentage decreased to 38 and 30% at T18 and T48, respectively (Figure 4C). However, while only about 30% of CD8+ lymphocytes were granzyme B+ at T0, the proportion of these activated/cytotoxic cells increased to 47 and 74% at T18 and T48, respectively (Figure 4C left).

We also detected an additional population represented by a double CD4+ CD8+ subset which displayed a kinetics similar to that of CD8+ T cells (Figure 4D). In fact, its relative amount progressively declined during therapy (Figure 4D left), whereas positivity for the granzyme B activation marker increased from 6% at T0 to 26% and 61% at T18 and T48, respectively (Figure 4D left).

With regard to the monocyte/macrophage population, CD163+ cells were well represented at T0, to slightly increase at T18 and to reduce strongly at T48 (Supplementary Figure 4). An increase in the percentage of CD163+ CD4+ cells was also observed from T0 to T48 whereas a small percentage of CD163+ CD8+/granzyme B+ sub-population was detected only at T18 (Supplementary Figure 4, left).

We also performed an analysis for other IC markers (Figure 4E). mIHC disclosed that all CD8+ lymphocytes expressed PD-1 at T0, while positivity for this marker strongly declined at T18 and T48 (Figure 4F), most likely reflecting PD-1 occupancy by nivolumab as observed in circulating lymphocytes (Figure 3A). We also observed a small amount of CD8+ PD-1+ lymphocytes that acquired LAG-3 expression after therapy (T18, T48; Figure 4F), while an additional subset of CD8+ PD-L1+ LAG3+ T cells appeared at T18 to decrease at T48 (Figure 4F). Only a minority of CD4+ T lymphocytes expressed PD-1 at T0, and this subset almost disappeared at T18 and T48 (Figure 4G). Furthermore, a CD4+ PD-L1+ LAG-3+ subset appeared at T18 to slightly reduce at T48 (Figure 4G). Finally, we did not observe any PD-L1 expression in SS cells both at T0 and T18 (data not shown).

## Discussion

SS has an unfavorable prognosis and current therapies are still unsatisfactory (12). Recent investigations have highlighted clinical benefits of PD-1 blockade in CTCL patients extensively pretreated (4–7). In particular, in a phase II study, pembrolizumab demonstrated significant antitumor activity (overall response rate of 38%) with durable responses and a favorable safety profile in patients with advanced Mycosis Fungoides/SS. However, the use of anti-PD-1 mAbs in SS is still debated since, secondary T-cell neoplasia in patients undergoing PD-1 blockade for their primary tumor, (9–11), as well as rapid disease progression in patients with T-cell leukemia/lymphoma treated with anti-PD-1 mAbs have also been documented (23–25). Understanding the effects of PD-1 blockade on blood and skin-resident SS cells and immune cell subsets during the course of therapy, hitherto not studied in detail, could be useful to evaluate SS patients’ response to PD-1 blockade, a therapeutic strategy under clinical investigation (ClinicalTrials.gov NCT03385226, NCT04118868).

Here, we report the results of a longitudinal analysis of circulating and skin-resident SS cells and immune sub-populations performed in a patient affected by metastatic melanoma and SS, who underwent nivolumab treatment for melanoma over a period of 44 weeks and showed a remarkable response of SS.

Our data show that nivolumab induced a progressive reduction in the absolute count/percentage of blood SS cells, which was accompanied by an increase in the percentage of normal CD4+ and CD8+ T cells and NK cells over total leukocytes. Notably, SS cell absolute count/percentage remained low even 16 weeks after therapy switching to dabrafenib + trametinib. Although we can not exclude that the targeted therapy was also effective on SS cells, the absence of BRAF mutations in SS cells (data not shown) does not support this possibility.

PD-1 is an exhaustion marker induced by chronic TCR engagement supporting an ongoing immune response (22). Instead, Ki67 expressed during cell proliferation, is a marker of anti-PD-1-induced T-cell invigoration of exhausted T cells (26). Previous studies conducted in melanoma patients treated with pembrolizumab demonstrated that the increase in Ki67 expression during therapy was higher in CD8+ PD-1+ than in CD8+ PD-1- PB lymphocytes (26). We therefore measured PD-1 expression in combination with Ki67 in patient’s immune sub-populations at T0 and T8. Consistent with the previous findings, the highest increase in the percentage of Ki67+ cells at T8 occurred in normal CD4+ T cells, followed by CD8+ T cells and NK cells, accordingly to their respective PD-1+ cell frequencies.

Recently, Saulite et al. (27) reported that PD-1 was expressed by SS cells and that *in vitro* treatment of PMA/ionomycin-stimulated SS cells with nivolumab enhanced their proliferation. In contrast, we observed that, although PD-1 was expressed by almost the totality of SS cells (92%) at T0, a 2-fold reduction in the percentage of Ki67+ cells occurred at T8, indicating that patient’s SS cells were not unlocked by nivolumab. It must be pointed out that our results were obtained on *ex vivo* SS cells and could be more indicative of nivolumab effects in the patient.

The skin compartment plays a crucial role in SS pathogenesis providing stimulatory signals for SS cell activation/proliferations (28) and contributing to immune evasion or immunosurveillance (29). IHC/mIHC conducted on lesional skin biopsies obtained at T0, T18 and T48 revealed a marked decrease in SS cells at T18 and their disappearance at T48 and an overall reduction in total lymphocyte density. Concurrently, we observed a progressive increase in the percentage of normal CD4+ T cells and a reduction in that of CD8+ and CD4+ CD8+ T-cell subsets from T0 to T48. However, a marked increase in granzyme B positivity was detected in CD8+ and CD4+ CD8+ T cells at T18 and T48, and to a lesser extent in CD4+ T cells at T18, suggesting a functional switching toward a cytotoxic/cytolytic activity (30).

These findings, together with the observation that all CD8+ T cells expressed PD-1 at T0, suggest that clearance of skin SS cells could result from an effective tumor-specific immune response induced by PD-1 blockade. A switching of the tumor microenvironment from an immune suppressive to a more reactive condition appears also supported by the finding that pro-tumorigenic M2 macrophages (CD163+ cells), although slightly increased at T18, appeared clearly reduced at T48.

Previous studies demonstrated that nivolumab is not able to mediate antibody-dependent cellular cytotoxicity and complement-mediated cytotoxicity (31). This rules out the possibility that a direct killing of PD-1+ SS cells could occur by these mechanisms. On the other hand, human NK cells can express PD-1, and PD-1 blockade can increase NK cell activity in the murine model (32). Moreover, SS cells have been previously reported to be target of activated autologous NK cells (33). It is, therefore, possible that the reduction of SS cells in the periphery and in skin tumor lesions could be, at least in part, dependent on nivolumab-mediated boosting of NK cell activity. Actually, in addition to the rise in NK cells which showed clear expression of PD-1 at T0 in peripheral blood, we also observed an increase of NK cells in the lesional skin at T18 with respect to T0 (data not shown).

PD-L1 expression in the tumor microenvironment by tumor and other cells elicits PD-1 signaling, and frequently associates with response to PD-1 blockade (34). At T0, we detected PD-L1 expression only in a small percentage of normal CD4+ T cells, while SS cells resulted PD-L1 negative. An increased percentage of normal CD4+ and CD8+ T cells expressing LAG-3 and PD-L1 was evidenced at T18, suggesting a compensatory mechanism of IC upregulation induced by PD-1 blockade. At T48, the frequencies of both T-cell sub-populations decreased, possibly as a result of nivolumab discontinuation. These findings indicate that, at least in our patient, the low expression of PD-L1 in SS cells and TILs, did not prevent a satisfactory clinical response to nivolumab.

An overall boosting of patient’s immune responses was also attested by the development of vitiligo-like skin lesions, an AE of IC inhibitors frequently observed in melanoma patients (35). This AE is associated with a favorable prognosis and depends on immune responses against antigens shared by melanoma and normal melanocytes (35). Presently, we can not exclude that immune response against some tumor antigens shared between SS cells and melanoma could have contributed to SS response to therapy. Interestingly, complete regression of Mycosis Fungoides, a low-grade CTCL (12, 13) was also observed in a patient with concurrent melanoma upon second-line therapy with the anti-PD-1 mAb pembrolizumab (36).

Although the present study and those of other authors (4–7) demonstrate a clear clinical benefit of PD-1 blockade in T-cell malignancies, it is important to underline that PD-1 is a potent haplo-insufficient tumor suppressor in T-cell lymphoma (8), and that its inhibition might potentially promote lymphomagenesis or accelerate neoplastic T cell growth. Actually, mono- and bi-allelic deletion of *PDCD1*, the gene coding for PD-1, have been detected in more than 30% of T-cell lymphomas (8) and rapid disease progression has been documented in some patients treated with anti-PD-1 mAbs for T-cell malignancies (23–25). Hyperprogression under anti-PD-1 mAbs has been reported also in patient with non-hematological tumors and several mechanisms have been implicated, including expansion of PD-1+ regulatory T cells (Tregs), compensatory up-regulation of alternative ICs, immunotherapy-related induction of cancer stem cells, reprogramming of tumor associated macrophage from M1 to M2 phenotype as a consequence of their binding to the Fc portion of the anti-PD-1 mAb (37). Those mechanisms could also underlie disease progression in a subset of patients with T-cell malignancies. Actually, expansion of tumor associated Tregs has been reported in Adult T-cell Leukemia/Lymphoma patients rapidly progressing on nivolumab (24). Interestingly, in melanoma patients not responding to PD-1 blockade, increased recruitment of Tregs in tumor microenvironment was associated with PTEN loss (38). Previous studies, including one by our group, demonstrated that PTEN deletion and/or epigenetic down-regulation occur frequently in SS (39) and hematological tumors (40). Moreover, *PDCD1* was found to be biallelically or heterozygously deleted in 5 and 15% of CTCLs, respectively (41), while focal deletion of 2q37.2, involving *PDCD1* and five other genes, has been identified in 36% of SS patients (42). It is possible to speculate that the genomic status of these tumor suppressor genes could, at least in part, underline the heterogenous responses T-cell neoplasias to anti-PD-1 mAbs.

## Conclusion

Nivolumab induced a remarkable clinical benefit for SS in our patient. Therapeutic efficacy might be also due to a good immunocompetence of this treatment-naïve patient. Our data also suggest that Ki67 expression in circulating neoplastic and immune cell subsets, as well as an enrichment in T cells with a cytotoxic phenotype in lesional skin could be valuable markers to assess early on treatment SS patients’ response to PD-1 blockade.

## Data Availability Statement

All datasets presented in this study are included in the article/Supplementary Material.

## Ethics Statement

The studies involving human participants were reviewed and approved by the Ethical Committee of the IDI-IRCCS (ID n. 4/CE/2015). The patients/participants provided their written informed consent to participate in this study. Written informed consent was obtained from the individual(s) for the publication of any potentially identifiable images or data included in this article.

## Author Contributions

AM, MA, MC, GAC, and FD were involved in patient care and clinical follow-up. FP evaluated pathologic skin specimens. LB processed an cryopreserved patient’s blood samples. AF and ES performed the flow cytometry analysis for clinical routine including TCR Vβ analysis. AT and AR performed the multiple immunofluorescence analysis on skin specimens. MP performed the flow cytometry for characterization of SS cells and immune cell subsets. MN, MP, GR, and SD analyzed the data and wrote the manuscript. All authors contributed to the article and approved the submitted version.

## Conflict of Interest

The authors declare that the research was conducted in the absence of any commercial or financial relationships that could be construed as a potential conflict of interest.
