# Supplementary material for: Reduction of T Lymphoma Cells and Immunological Invigoration in a Patient Concurrently Affected by Melanoma and Sezary Syndrome Treated With Nivolumab
Source: Front Immunol. 2020 Sep 25;11:579894. doi: 10.3389/fimmu.2020.579894 (PMC7544958; doi:10.3389/fimmu.2020.579894)
Supplement: Supplementary file 1 [file Data_Sheet_1.PDF]

Supplementary Information

Material and methods

Flow cytometry (FC) analysis for clinical routine.

Percentages and absolute counts of T, B, and natural killer (NK) cells, as well as those of the CD4+ and CD8+ subpopulations of circulating T cells were determined by the Multitest 6-color TBNK reagent (BD Bioscience, San Jose, CA) containing the anti-CD3-FITC, anti-CD16-PE + anti-CD56 PE, anti-CD45-PerCP-Cy5.5, anti-CD4 PE-Cy7, anti-CD19-APC, and anti-CD8-APC-Cy7 mAbs, using a lyse-no wash EDTA-whole blood protocol according to the manufacturer's instructions. The stained samples were acquired using a BD FACSCanto II cytometer (BD Biosciences) and immune population analyzed with the BD FACSCanto clinical software using a CD45 (CD45 *vs* SSC) gating strategy to identify total lymphocytes.

Absolute counts of patient’s neoplastic lymphocytes (i.e SS cells) were measured by detection of their specific TCR-Vβ 5.1 rearrangement using the mix C of a panel of 23 mAbs (IO Test beta mark TCR Vβ repertoire kit, Beckman Coulter, Fullerton, CA), as described previously (1) in combination with anti-CD4-PerCP mAb (BD Bioscience). Cells were analyzed on a BD FACSCanto II cytometer using the BD FACSDiva software with a 3-color setting protocol.

FC analysis for characterization of SS cells and immune cell subsets

FC on a CytoFlex LX machine (Beckman Coulter) was used to evaluate in depth the effects of nivolumab on neoplastic cells and immune cell subsets. Peripheral blood mononuclear cell (PBMC) preparations were stained in V-bottom 96-well plates for 15 minutes at room temperature with antibodies diluted in a final volume of 30 μl/well of BD Brilliant Stain Buffer. FoxP3 buffer set (ThermoFisher, Waltham, MA) was used to detect intracellular markers after surface staining as described (1). Mix C of IO Test Beta Mark TCR Vβ repertoire kit was used to count clonal TCR-Vβ5.1+ tumor cells, whereas mAbs against CD3, CD4, CD8, CD16, CD19, CD71, Ki67, HLA-DR, and PD-1 markers, described in detail in Supplementary Table S2, were employed for characterization of immune cell subsets and SS cells. Compensation and gating were performed by the FlowJo software (BD Bioscence).

Immunohistochemistry (IHC) and multiplex fluorescence IHC (mIHC)

Diagnosis of CTCL was performed by routine histopathological analysis of a lesional skin biopsy collected at disease onset that was further confirmed by TCR clonality assessment by PCR-BIOMED-2 (2). IHC for CD3, CD4 and CD8 markers was carried out on 4 μm-thick sections of formalin-fixed paraffin-embedded (FFPE) skin biopsies obtained from patient at T0 and T18, as previously described (1). FFPE skin biopsies obtained at T0, T18, plus an additional one collected 4 weeks after the end of nivolumab treatment and therapy switching to dabrafenib+ trametinib (T48) were subjected to 7-color mIHC using the Opal 7-colors manual IHC kit (Akoya Biosciences, Marlborough, MA). To characterize the subsets of neoplastic cells and tumor infiltrating lymphocytes (TILs) we used two

staining panels (Supplementary Table S3). The first one included mAbs against TCR Vβ5.1 - to specifically identify neoplastic CD4+ SS cells, LAG-3, CD4, CD8, granzyme B and CD163. The second one included mAbs against TCR Vβ5.1, CD4, CD8, PD-1, LAG-3 and PD-L1. Antigen retrievals were performed in a microwave oven using Target Retrieval Solution pH9 (Agilent Technologies, Santa Clara, CA) or pH6 (Akoya Biosciences), respectively. The staining procedure consisted of sequential rounds of protein blocking with Protein Block Serum-free (Agilent Technologies), followed by primary antibody and secondary Horseradish Peroxidase-conjugated antibody that mediates the covalent binding of a different Tyramide Signal Amplification -conjugated Opal fluorophore (Akoya Biosciences) to the antigen. DAPI was used to counterstain nuclei. Multiplex stained slides were scanned at 20X using the Mantra Quantitative Pathology Workstation (Akoya Biosciences), and analyzed with InForm Image Analysis Software (Akoya Biosciences v2.4.2).

Supplementary Tables

Supplementary Table 1. Changes of CD4+/CD8+ T cell ratio during nivolumab therapy

CD4+/CD8+ Tcell ratio*	Weeks from the start of nivolumab
14.6	T0
9.4	T2
13.5	T4
4.5	T8
7.2	T18
6.4	T24
4.8	T34
3.7	T42

*Ratio of absolute counts (cells/μl blood) determined by routine clinical analyses

Supplementary Table 2. List of mAbs used for SS cell and immune cell subset characterization by flow cytometry

Antigen	Fluorochrome	Clone	Vendor	Locality
CD3	BUV395	UCHT1	BD biosciences	Franklin Lake, NJ
CD4	PerCP-Cy5.5	RPA-T4	Biolegend	San Diego, CA
CD8	CD8 BV605	HIT8a	BD biosciences	Franklin Lake, NJ
CD16	BUV496	3G8	BD biosciences	Franklin Lake, NJ
CD19	PE-Cy5.5	J3-119	Coulter	Brea, CA
CD71	FITC	CY1G4	Biolegend	San Diego, CA
Ki-67	APC	Ki-67	Biolegend	San Diego, CA
HLA-DR	BV786	G46-6	BD biosciences	Franklin Lake, NJ
PD-1	BV650	EH12.1	BD biosciences	Franklin Lake, NJ
Live/dead	Aqua	NA	ThermoFisher	Waltham, MA

Supplementary Table 3. List of mAbs used for mIHC analysis

Panel 1				
**Antigen**	**Fluorochrome**	**Clone**	**Vendor**	**Locality**
TCR Vβ5.1	Opal 520	IMMU 157	Beckman Coulter	Brea, CA
Granzyme B	Opal 540	11F1	Leica Biosystems	Wetzlar, Germany
CD4	Opal 620	4B12	Thermo Fisher Scientific	Waltham, MA
CD8	Opal 690	C8/114B	Agilent Technologies	Santa Clara, CA -
CD163	Opal 570	10D6	Leica Biosystems	Wetzlar, Germany
Nuclei	Spectral DAPI		Akoya Biosciences	Marlborough, MA

Panel 2				
**Antigen**	**Fluorochrome**	**Clone**	**Vendor**	**Locality**
TCR Vβ5.1	Opal 520	IMMU 157	Beckman Coulter	Brea, CA -
LAG-3	Opal 570	17B4	Abcam	Cambridge,
CD4	Opal 620	4B12	Thermo Fisher Scientific	Waltham, MA,
CD8	Opal 690	C8/114B	Agilent Technologies	Santa Clara, CA
PD-1	Opal 650	EPR4877-2	Abcam	Cambridge, UK
PD-L1	Opal 540	E1L3N	Cell Signaling Technology	Danvers, MA
Nuclei	Spectral DAPI		Akoya Biosciences	Marlborough, MA

Supplementary Figures

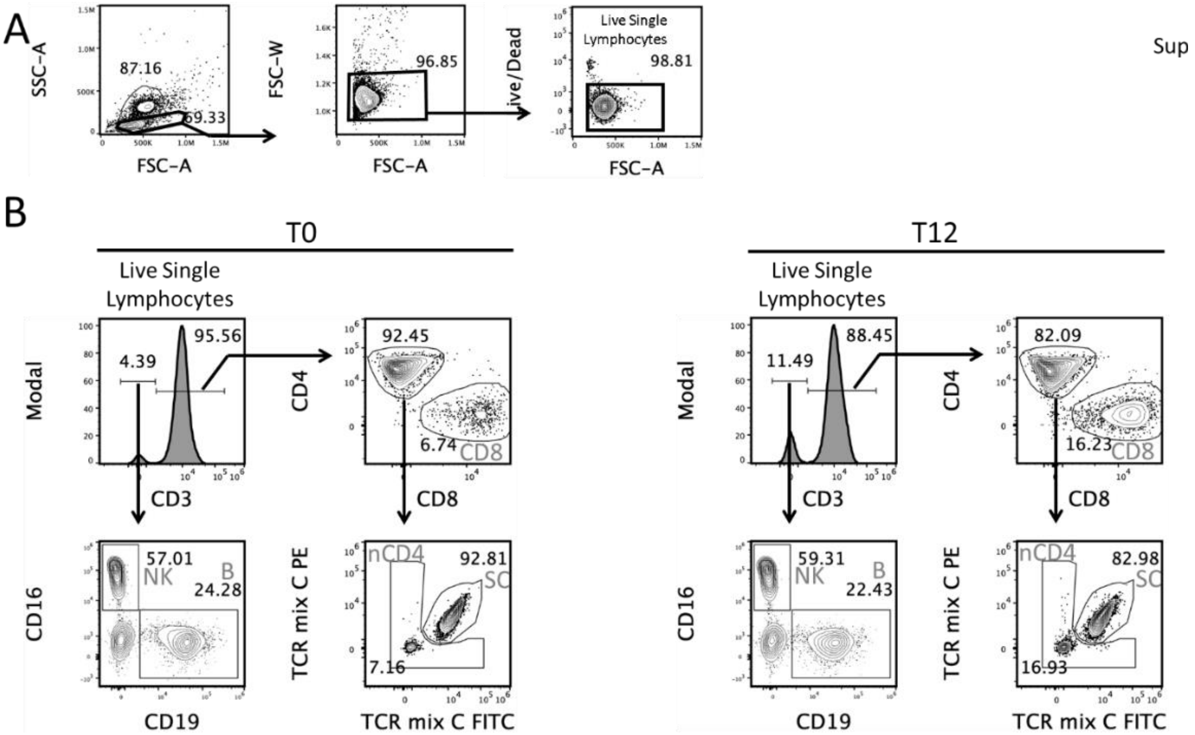

Supplementary Figure 1. Gating strategy used to identify major lymphocyte subsets. A, Filtering of live single lymphocytes. Sequential gating was routinely used to select lymphocytes with a wide gate on forward *vs* side scatter (FSC-A vs SSC-A) to include large SS cells, while doublets and dead cells were excluded by gating out events with large forward scatter pulse width (FSC-W) and positive for a viability stain. **B,** Selection of lymphocyte subsets performed in parallel for the T0 and T8 time-points. Numbers indicate frequencies of adjacent gates among parent populations

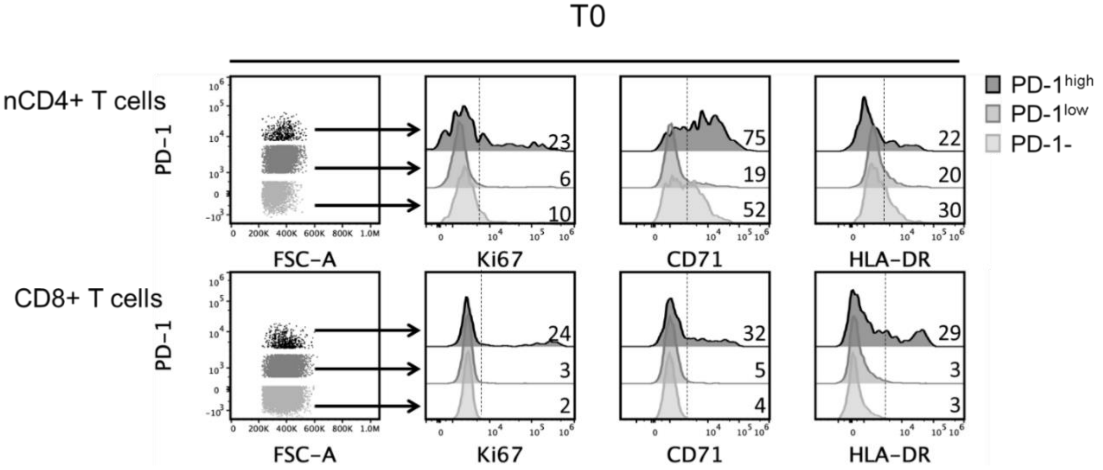

Supplementary Figure 2. Activation markers in PD-1 expression-related subsets. Normal (n) CD4+ and CD8+ T cells obtained at T0 were stained with anti-PD-1 in combination with anti-Ki67, anti-CD71 and anti-HLA-DR mAbs and sub-gated by PD-1 expression intensity. Ki67+, CD71+ and HLA-DR+ cell frequencies are indicated for each nCD4+and CD8+ T cell subsets into the histograms.

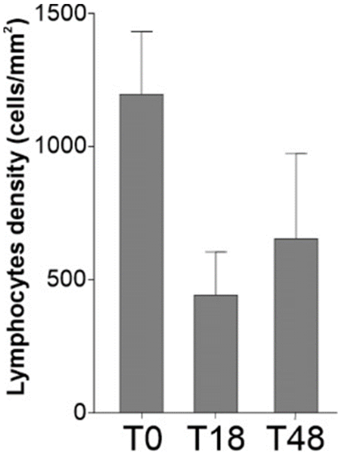

Supplementary Figure S3. Density (cells/mm^2) of total lymphocytes infiltrating biopsies collected at T0, T18 and T48. Mean values and standard deviation (SD) derived from the analysis of the same fields considered in Figures 4B-D

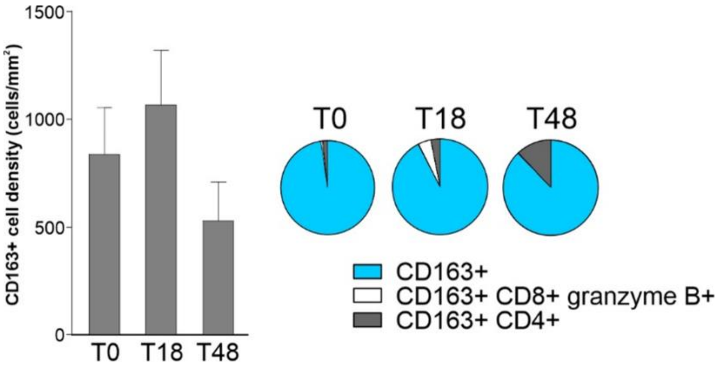

Supplementary Figure 4. CD163+ cell density. Left: CD163+ cell density (cells/mm^2) in biopsies collected at T0, T18 and T48. Data reported for each cell subset are the mean values and SD of the same fields considered in Figures 4B-D. Right: pie charts of mIHC data from biopsies collected at T0, T18 and T48. Data reported for each cell subset are the mean values derived from the analysis of the same fields considered in Figures 4B-D and in the flanking histograms.

<u>References</u>

1. Cristofoletti C, Bresin A, Picozza M, Picchio MC, Monzo F, Helmer Citterich M, Passarelli F, Frezzolini A, Scala E, Monopoli A, et al. Blood and skin-derived Sezary cells: differences in proliferation-index, activation of PI3K/AKT/mTORC1 pathway and its prognostic relevance. *Leukemia* (2018) doi:10.1038/s41375-018-0305-8

2. van Dongen JJM, Langerak AW, Brüggemann M, Evans PAS, Hummel M, Lavender FL, Delabesse E, Davi F, Schuuring E, García-Sanz R, et al. Design and standardization of PCR primers and protocols for detection of clonal immunoglobulin and T-cell receptor gene recombinations in suspect lymphoproliferations: Report of the BIOMED-2 concerted action BMH4-CT98-3936. *Leukemia* (2003) **17**:2257–2317. doi:10.1038/sj.leu.2403202
